# Associations between dyslexia and mental health in primary school students: the roles of parenting style, and character strengths

**DOI:** 10.1080/00049530.2026.2669142

**Published:** 2026-05-07

**Authors:** Weiyan Feng, Hongyu Liu, Kyaw Ko Ko Htet, Rassamee Chotipanvithayakul

**Affiliations:** a Ningxia Vocational and Technical College for Minorities, Wuzhong, China; b Department of Epidemiology, Faculty of Medicine, Prince of Songkla University, Hat Yai, Thailand; c School of Humanities and Health Management, Ningxia Medical University, Yinchuan, China; d Research Center for Child and Youth Development, Faculty of Medicine, Prince of Songkla University, Hat Yai, Thailand

**Keywords:** Dyslexia, parenting style, character strengths, anxiety, depression

## Abstract

**Objective:**

Dyslexia, a neurodevelopmental disorder that impairs reading, poses risks to students’ academic and psychological well-being. This study examined the associations between dyslexia and mental health outcomes (anxiety and depression) in primary school students, focusing on the moderating roles of parenting style, and character strengths.

**Method:**

A cross-sectional survey was conducted among 2,322 primary school students in Yinchuan city, China. Dyslexia risk was identified through multi-step screening. Binary logistic regression was used to test main effects and interaction effects (dyslexia × parenting style, dyslexia × character strengths), adjusting for sociodemographic covariates.

**Results:**

Dyslexia risk was identified in 137 students (5.9%). In main-effect models, dyslexia risk was associated with higher depression (OR = 1.88, 95% CI: 1.23–2.86, *p* = .003) but not anxiety (*p* = .190). Paternal overprotection significantly moderated the relationship between dyslexia and depression (OR for interaction = 0.16, *p* = .011), while no significant moderation was found for anxiety or for character strengths. These findings suggest that the mental health impact of dyslexia is context-dependent rather than uniformly buffered by protective factors.

**Conclusions:**

Paternal overprotection specifically exacerbates depression risk among children with dyslexia, whereas general protective effects (e.g. emotional warmth) apply similarly to all children. Interventions should target parenting practices in families of children with reading difficulties.

## KEY POINTS

What is already known about this topic:

(1) Dyslexia increases mental health risks: Students with dyslexia often experience higher anxiety and depression than their peers, reflecting the broader psychosocial impact of reading difficulties.

(2) Parenting styles play a critical role in children’s socioemotional adjustment, with emotionally warm parenting linked to better psychological outcomes.

(3) Character strengths have been identified as protective factors that promote resilience and well-being in general student populations.

What this topic adds:

(1) This study distinguishes between general protective effects and dyslexia-specific moderation: paternal overprotection exacerbates depression specifically in children with dyslexia.

(2) No evidence was found that character strengths moderate the association between dyslexia and mental health, suggesting their benefits are universal rather than compensatory.

(3) The moderating effect of overprotective parenting is specific to fathers, not mothers, highlighting the distinct roles of paternal and maternal parenting in Chinese families.

(4) Dyslexia is associated with depression but not anxiety, suggesting that academic difficulties may differentially impact internalizing problems.

## Introduction

Dyslexia is a neurodevelopmental disorder characterized by specific learning disabilities such as slow reading and inaccurate word recognition. Dyslexia affects approximately 7% of primary students in China and worldwide (R. L. Peterson & Pennington, 2012; Yang et al., 2022). Recent studies have underscored the heightened vulnerability of students with dyslexia not only to academic challenges but also to mental health issues. These challenges often manifest as increased risks of depression and anxiety (Xiao et al., 2022), thereby impacting their overall well-being.

These findings underscore that the implications of dyslexia extend beyond literacy skills, raising critical questions for educational psychology regarding the processes through which learning difficulties shape students’ broader developmental and mental health outcomes.

Educational psychology frameworks highlight that children’s socioemotional adjustment is shaped not only by cognitive skills but also by their proximal social and motivational environments. Parenting, for example, plays a central role in shaping students’ psychological outcomes and may represent a key pathway through which dyslexia influences mental health. Consistent with attachment theory, emotionally warm parenting fosters secure relationships, reduces perceived stress, and promotes adaptive emotion regulation (Bowlby, 1988; Mikulincer & Shaver, 2007; Thompson, 1994). In contrast, rejection or overprotection has been linked to heightened risk of depression and anxiety. Warm parenting also supports the development of positive self-concept, resilience, and character development (Asbari et al., 2020) among children. These findings suggest that parenting may represent one pathway through which the experience of dyslexia influences mental health outcomes. While parenting represents a critical environmental influence, individual psychological resources also contribute to children’s resilience.

Complementing these family influences, individual-level protective factors such as character strengths (CS) – defined as positive psychological traits that support resilience and flourishing (C. Peterson & Seligman, 2004) – are increasingly recognized as critical in educational contexts. Character strengths foster adaptive coping, intrinsic motivation, and psychological adjustment (Park & Peterson, 2006; Waters, 2011). For students with dyslexia, persistent academic struggles may undermine the development of certain strengths, yet other moral-related strengths such as fairness or humility may be relatively preserved (Feng et al., 2024). By buffering against stress and reinforcing self-worth, character strengths may function as mediators in the association between dyslexia and mental health. This aligns with positive education perspectives that emphasize building on students’ strengths as a foundation for resilience and well-being.

Despite growing evidence in general populations, little is known about how psychosocial and behavioural processes operate in children with dyslexia, particularly in educational contexts with limited awareness and support, such as in China. Parenting style comprises multiple dimensions, including emotional warmth, parental anxiety, rejection, and overprotection, all of which may shape children’s psychological outcomes.

To address these gaps in knowledge, the present study examined whether parenting style and character strengths moderate the association between dyslexia and anxiety/depression. By testing interaction effects (Dyslexia × Parenting style, Dyslexia × Character Strengths), we aimed to determine whether these factors confer general benefits or specifically buffer mental health risks for children with dyslexia. This approach provides clearer evidence for targeted interventions in under-recognized contexts such as China.

## Materials and methods

### Study setting and participants

This study was conducted in 2023 in public primary schools in Yinchuan city, northwest China. Nine schools were randomly selected from 203 schools. From each selected school, one classroom per grade (Grades 2 to 6; ages 8–12) was randomly selected, and all enrolled students within these classrooms and their parents were eligible for inclusion. Grade 1 students were excluded due to their limited reading instruction and difficulty completing self-reports. The exclusion criteria included a history of brain injury, visual or auditory disorders, epilepsy, and other neurological conditions.

### Measures

#### Sociodemographic

Students and parents completed questionnaires to collect sociodemographic data, including the student’s grade, gender, parental age, education and occupation, and household monthly income.

#### Parenting style

Perceived parenting was assessed using the Egna Minnen Beträffande Uppfostran-Children (EMBU-C) questionnaire (Arrindell et al., 1999). The four subscales – emotional warmth, anxiety, rejection, and overprotection – were used, with Cronbach’s α = 0.74–0.82 for the validated Chinese version.

#### Character strengths

CS were assessed using the VIA Youth Survey (Miller et al., 2021), which includes 103 items across 24 subscales for ages 8–13. Reliability ranged from α = 0.60–0.90. Higher mean scores indicated stronger CS. The mean scores were used as continuous variables in the logistic regression analyses.

#### Mental health outcomes

Depression and anxiety were assessed using the Children’s Depression Inventory (CDI) (Kovacs, 2015), the Screen for Child Anxiety Related Emotional Disorders (SCARED) (Su et al., 2008), respectively. The CDI Chinese version has demonstrated good validity and reliability (Cronbach’s α = 0.70–0.87), with a cut-off of 20 for depressive symptoms (Matthey & Petrovski, 2002). The SCARED comprises 41 items rated 0–2, with scores > 30 indicating clinically significant anxiety. Internal consistency in Chinese samples ranged from α = 0.43–0.89.

#### Dyslexia risk screening

All 2nd to 6th grade students underwent a multi-step dyslexia screening procedure, consistent with commonly used practices in China. Students with IQ < 75 (American Psychiatric Association, 2013), assessed using the Combined Raven’s Test (CRT) (Li et al., 1988), were first excluded. Among the remaining students, those whose Chinese language test scores were in the lowest 20% of their class were selected for further evaluation. These students then completed the Pupil Rating Scale Revised (PRS) (Jing et al., 1995) and the Dyslexia Checklist for Chinese Children (DCCC) (Hou et al., 2018). Students were classified as having dyslexia risk if they met both of the following criteria: PRS score < 65 and DCCC score > 2 times standard deviations above the mean. Both PRS and DCCC have demonstrated high reliability, with test-retest coefficients > 0.80 for PRS and Cronbach’s α = 0.97 for DCCC (Cai et al., 2020).

### Data collection

The study was approved by the Institutional Ethics Committee of the Faculty of Medicine, Prince of Songkla University, Thailand (Protocol No. 65-331-18-9). Data collection started after approval was received from the school principals. Prior to participation, researchers distributed a detailed study information sheet and informed consent form to students, who were instructed to take them home. Parents were asked to review the materials and provide written informed consent for both their own and their child’s participation in the study.

On the day of data collection, the researchers also gave an age-appropriate verbal explanation to the participating students, outlining the study’s objectives, procedures, and their rights. Written assent was obtained from each student. Only those students whose parents had signed the consent form and who themselves provided written assent were included in the study.

The voluntary nature of participation was emphasized throughout the process, and all participants were informed that they could withdraw from the study at any time without any penalty or consequences.

The researchers distributed a set of self-response questionnaires that included the CRT, EMBU-C, VIA Youth Survey, CDI, and SCARED, to each child to complete in the classroom. The researchers attended the class to provide assistance as needed and ensured that the students completed the questionnaires independently. Two 10-minute breaks were provided. Completed questionnaires were sealed in envelopes and returned to the researchers. Subsequently, the Chinese language teachers screened for dyslexia using the PRS and DCCC scales.

The self-administered questionnaire was delivered to parents by their child. After completion, the parents sealed the questionnaire in an envelope and returned it to the teacher the following day or within 3 days. The researchers collected the envelopes within one week after that.

### Statistical analysis

#### Data management and analysis

Data entry and validation were performed using EpiData 3.1 (Lauritsen et al., 2003). Statistical analyses were conducted using R (version 4.2.2). Descriptive statistics were calculated for all variables. Group differences between students with and without dyslexia were examined using χ^2^ tests for categorical variables and t-tests for continuous variables.

Binary logistic regression analyses were performed to examine the associations between dyslexia and mental health outcomes (depression and anxiety). Separate models were constructed for each outcome. Dyslexia status was included as the main independent variable. Parenting style, and character strengths were then added to the models to assess their associations with mental health outcomes. To test moderation effects, we added interaction terms (Dyslexia × Parenting Style, Dyslexia × Character Strengths) to the models. Model fit was compared using likelihood-ratio tests and AIC values. Multivariable models adjusted for potential confounders, including gender, school grade, parental age, parental education, occupation, and household monthly income. Adjusted odds ratios (ORs) with 95% confidence intervals (CIs) were reported. Statistical significance was set at *p* < 0.05.

## Results

Out of 2,549 students and their parents who were invited to participate, 2,443 agreed to participate (95.8% response rate). Subsequently, the students were screened for dyslexia. A total of 121 students were excluded due to absence (*n* = 52), IQ < 75 (*n* = 17), and whose questionnaire completion rate was less than 90% on either the student’s or parent’s questionnaire (*n* = 52), the final sample comprised 2,322 students. Among them, 137 (5.9%, 95% CI: [4.9%, 6.9%]) were identified as having dyslexia.

### Comparisons of various variables and mental health outcomes between students with and without dyslexia

Table 1 presents the sociodemographic characteristics of students with and without dyslexia. Males were more prevalent among students with dyslexia. Significant differences were observed in paternal education and household monthly income, which were lower in the dyslexia group, whereas no differences were found in school grade, maternal education, parental age, or occupation.

**Table 1.** Sociodemographic characteristics between students with and without dyslexia (*N* = 2,322).

	Dyslexia *n* = 137	Non-dyslexia *n* = 2,185	*p*-value
Gender			<0.001
Female	38 (27.7)	1,109 (50.8)	
Male	99 (72.3)	1,076 (49.2)	
School grade			0.427
2nd	32 (23.4)	439 (20.1)	
3rd	30 (21.9)	469 (21.5)	
4th	31 (22.6)	471 (21.6)	
5th	26 (19.0)	376 (17.2)	
6th	18 (13.1)	430 (19.7)	
Maternal age, mean (SD)	35.5 (5.0)	35.7 (4.9)	0.610
Paternal age, mean (SD)	38 (5.0)	38.2 (5.4)	0.668
Maternal education level			0.074
Junior high school or below	89 (65.0)	1,226 (56.1)	
Senior high school or junior college or equivalent	28 (20.4)	477 (21.8)	
Junior college or above	20 (14.6)	482 (22.1)	
Paternal education level			0.015
Junior high school or below	84 (61.3)	1,178 (53.9)	
Senior high school or junior college or equivalent	35 (25.5)	485 (22.2)	
Junior college or above	18 (13.2)	522 (23.9)	
Maternal occupation			0.211
Farming, forestry, fishery worker	22 (16.1)	264 (12.1)	
Business or staff in institution or government*	39 (28.5)	753 (34.5)	
Others	76 (55.5)	1,168 (53.5)	
Paternal occupation			0.162
Farming, forestry, fishery worker	21 (15.3)	251 (11.5)	
Business or staff in institution or government*	51 (37.2)	977 (44.7)	
Others	65 (47.4)	957 (43.8)	
Household monthly income (RMB)**			<0.001
≤5,000	92 (67.2)	1,087 (49.7)	
>5,000 to <15,000	38 (27.7)	962 (44.0)	
≥15,000	7 (5.1)	136 (6.2)	

Data are shown as *n* (%) unless otherwise indicated.

*Including professional technical staff, principals of institutions or government, office staff, service staff, business staff, production worker, transport worker, and other related occupations. **1 RMB ≅ 0.14 USD.

Table 2 presents parenting styles, character strengths, and mental health outcomes. Students with dyslexia exhibited lower emotional warmth and approximately twice the levels of rejection or overprotection parenting style compared to their peers. Students with dyslexia showed higher prevalence of depression (36.5%), whereas no significant differences were observed in anxiety symptoms.

**Table 2.** Parenting style, CS, and mental health outcomes between students with and without dyslexia (*N* = 2,322).

	Dyslexia *n* = 137	Non-dyslexia *n* = 2,185	*p*-value
Parenting style			
Maternal parenting style			0.124
Emotional warmth	85 (62.0)	1,515 (69.3)	
Anxious	31 (22.6)	457 (20.9)	
Rejection	10 (7.30)	86 (3.9)	
Overprotection	11 (8.0)	127 (5.8)	
Paternal parenting style			0.004
Emotional warmth	80 (58.4)	1,518 (69.5)	
Anxious	28 (20.4)	424 (19.4)	
Rejection	15 (11.0)	124 (5.7)	
Overprotection	14 (10.2)	119 (5.5)	
Character strengths, mean (SD)	3.4 (0.5)	3.4 (0.4)	0.147
Mental health			
Depression symptoms			<0.001
Yes	50 (36.5)	439 (20.1)	
No	87 (63.5)	1746 (79.9)	
Anxiety disorder symptoms			0.103
Yes	36 (26.3)	439 (20.1)	
No	101 (73.7)	1746 (79.9)	

Data are reported as *n* (%) unless otherwise indicated. The identical counts for depression and anxiety in the non-dyslexia group (439 each) are coincidental. These represent the number of children meeting the clinical cut-off for each condition separately; the two groups are not identical, and 223 children (10.2%) meet criteria for both conditions.

### Main effect of dyslexia, parenting style and character strengths on mental health

Binary logistic regression was conducted to examine the main effects of dyslexia status, parenting styles (paternal and maternal: emotional warmth, anxious, overprotection, rejection), and character strengths on depression and anxiety, adjusting for gender, grade, parental age, education, occupation, and household income. Variance inflation factors (VIF) for all predictors were below 3.0, indicating no multicollinearity concerns.

#### Depression

As shown in Table 3, dyslexia risk was significantly associated with higher odds of depression (OR = 1.88, 95% CI: [1.23, 2.86], *p* = .003). All maladaptive parenting styles were independently associated with increased depression risk. Specifically, paternal anxious parenting (OR = 1.93, 95% CI: [1.38, 2.70], *p* < .001), paternal overprotection (OR = 3.75, 95% CI: [2.26, 6.21], *p* < .001), and paternal rejection (OR = 4.34, 95% CI: [2.80, 6.73], *p* < .001) showed strong effects. Similarly, maternal anxious parenting (OR = 1.81, 95% CI: [1.30, 2.51], *p* < .001), maternal overprotection (OR = 1.63, 95% CI: [0.98, 2.68], *p* = .057, marginally significant), and maternal rejection (OR = 5.52, 95% CI: [3.28, 9.36], *p* < .001) were associated with higher depression. Character strengths were strongly protective (OR = 0.62 per unit increase, 95% CI: [0.55, 0.70], *p* < .001). The full model showed adequate fit (AIC = 2036.6).

**Table 3.** Main effects logistic regression for depression (*N* = 2,322).

Predictor	OR	95% CI	*p*-value
Dyslexia status (yes)	1.88	[1.24, 2.87]	.003
Paternal parenting (ref = emotional warmth)			
Anxious	1.93	[1.38, 2.70]	<.001
Overprotection	3.75	[2.26, 6.21]	<.001
Rejection	4.34	[2.80, 6.73]	<.001
Maternal parenting (ref = emotional warmth)			
Anxious	1.81	[1.30, 2.51]	<.001
Overprotection	1.63	[0.98, 2.68]	.058
Rejection	5.52	[3.28, 9.36]	<.001
Character strengths (per unit)	0.62	[0.55, 0.70]	<.001

OR = odds ratio; CI = confidence interval. Adjusted for gender, grade, parental age, education, occupation, and household income. Model AIC = 2036.6.

#### Anxiety

For anxiety (Table 4), dyslexia status was not a significant predictor (OR = 1.32, 95% CI: [0.86, 2.00], *p* = .190). Significant parenting predictors included paternal anxious parenting (OR = 1.85, 95% CI: [1.34, 2.56], *p* < .001), paternal rejection (OR = 2.84, 95% CI: [1.83, 4.36], *p* < .001), maternal anxious parenting (OR = 1.49, 95% CI: [1.08, 2.04], *p* = .015), and maternal rejection (OR = 3.25, 95% CI: [2.00, 5.30], *p* < .001). Paternal overprotection (*p* = .466) and maternal overprotection (*p* = .242) were not significant. Character strengths showed a protective trend but did not reach significance (OR = 0.93, 95% CI: [0.84, 1.03], *p* = .172). The full model showed adequate fit (AIC = 2252.7).

**Table 4.** Main effects logistic regression for anxiety (*N* = 2,322).

Predictor	OR	95% CI	*p*-value
**Dyslexia status (yes)**	1.32	[0.86, 2.00]	.190
**Paternal parenting (ref = emotional warmth)**			
Anxious	1.85	[1.34, 2.56]	<.001
Overprotection	1.23	[0.71, 2.13]	.466
Rejection	2.84	[1.83, 4.36]	<.001
**Maternal parenting** (ref = emotional warmth)			
Anxious	1.49	[1.08, 2.04]	.015
Overprotection	1.36	[0.81, 2.28]	.242
Rejection	3.25	[2.00, 5.30]	<.001
**Character strengths (per unit)**	0.93	[0.84, 1.03]	.172

OR = odds ratio; CI = confidence interval. Adjusted for gender, grade, parental age, education, occupation, and household income. Model AIC = 2252.7.

### Interaction effects: testing parenting style and character strengths as the moderators of the dyslexia – mental health relationship

To determine whether parenting styles specifically moderate the association between dyslexia and mental health (i.e., whether the effect of parenting differs between children with and without dyslexia), we conducted separate interaction models. Model fit was compared using likelihood-ratio tests and AIC values.

#### Depression

The interaction model, adding the dyslexia status and paternal parenting style interaction terms, significantly improved model fit over the main-effects model (χ^2^(3) = 9.43, *p* = .024; AIC improved from 2036.6 to 2033.2). The interaction Dyslexia × Paternal overprotection was statistically significant (OR = 0.16, 95% CI: [0.04, 0.65], *p* = .011). The interaction Dyslexia × Paternal rejection showed a trend towards significance (OR = 0.31, 95% CI: [0.08, 1.19], *p* = .084), while Dyslexia × Paternal anxious was not significant (*p* = .833).

In contrast, the interaction model with maternal parenting styles did not improve model fit compared to the main-effects model (χ^2^(3) = 0.49, *p* = .920; AIC: 2042.1 vs. 2036.6 for main model). None of the individual interaction terms (Dyslexia × Maternal anxious, overprotection, or rejection) reached statistical significance (all *p* > .60). Thus, maternal parenting did not significantly moderate the dyslexia–depression relationship.

The interaction between dyslexia status and character strengths was not significant (OR = 1.27, 95% CI: [0.89, 1.82], *p* = .188), and the interaction model did not improve fit over the main-effects model (χ^2^(1) = 1.68, *p* = .195; AIC: 2036.9 vs. 2036.6). This suggests that character strengths confer a general protective effect against depression that does not differ by dyslexia status.

#### Anxiety

For anxiety outcomes, none of the interaction models showed significant improvement over main-effects models: Dyslexia × Paternal parenting (χ^2^(3) = 1.80, *p* = .615; AIC: 2256.9 vs. 2252.7), Dyslexia × Maternal parenting (χ^2^(3) = 0.40, *p* = .940; AIC: 2258.3 vs. 2252.7), or Dyslexia × Character strengths (χ^2^(1) = 2.20, *p* = .138; AIC: 2252.5 vs. 2252.7). All individual interaction terms were non-significant (all *p* > .10).

## Discussion

This study investigated whether parenting style and character strengths moderate the association between dyslexia status and mental health outcomes among primary school students, using binary logistic regression with interaction terms. Consistent with previous findings, dyslexia risk was associated with higher depression but no direct association was observed with anxiety (Xiao et al., 2022). However, the key novel finding is that paternal overprotection significantly moderated the dyslexia–depression relationship (OR for interaction = 0.16, *p* = .011), indicating that the effect of overprotective parenting on depression differs between children with and without dyslexia. In contrast, maternal parenting and character strengths did not show significant interaction effects, suggesting they confer general protective or risk effects that operate similarly across both groups.

Consistent with previous research (Xiao et al., 2022), dyslexia risk was positively associated with depression, suggesting that persistent academic difficulties may heighten vulnerability to internalising problems. Extending this finding, we found that paternal overprotection significantly moderated this association. The significant interaction indicates that the association between paternal overprotection and depression differs significantly between children with and without dyslexia. This pattern suggests that children with dyslexia may experience a ceiling effect of depression risk, where multiple risk factors (dyslexia itself plus overprotection) do not combine additively but rather show a complex interaction pattern.

The absence of significant interactions for maternal parenting is noteworthy. This may reflect cultural norms in Chinese families, where fathers are often more involved in academic monitoring and discipline (Shek & Lee, 2007; Wen et al., 2024), making their overprotective behaviours particularly salient for children with reading difficulties.

Emotional warmth in parenting emerged as a key protective mechanism in main-effect models. Children who perceived their parents as emotionally supportive reported lower levels of depression and anxiety, highlighting the role of secure emotional relationships in mitigating psychological distress. Crucially, this effect did not interact with dyslexia status, indicating that emotional warmth is universally beneficial across both groups. This pattern is consistent with attachment theory (Bowlby, 1988; Mikulincer & Shaver, 2007; Thompson, 1994), which posits that emotionally secure environments foster effective emotion regulation and adaptive coping. Within the Chinese educational context – where academic achievement is highly valued and dyslexia often remains under-recognised – warm parenting may provide a critical buffer against both academic stress and negative affect.

Character strengths also played an important role in promoting socioemotional adjustment. Students with higher character strengths demonstrated lower depressive symptoms in main-effect models, and this effect did not differ by dyslexia status (interaction *p* = .188), suggesting that positive psychological traits facilitate adaptive coping in the face of academic challenges for all children. This aligns with the principles of positive education (Park & Peterson, 2006; Peterson & Seligman, 2004), which emphasise cultivating personal strengths as pathways to resilience and well-being. Prior studies have shown that strengths such as hope, zest, and self-regulation could reduce depression in adults (Proyer et al., 2015). Appreciation of beauty and excellence, love, perseverance, social intelligence, zest, perspective, and creativity help reduce anxiety in early adolescents (Qin et al., 2022). Our previous study (Feng et al., 2024) found that students with dyslexia scored lower in perseverance compared to peers, suggesting potential for growth. Strengths that facilitate academic achievement – such as prudence, self-regulation, hope, love of learning, and creativity – may be particularly valuable. Even in the context of dyslexia, cultivating or leveraging existing strengths appears to buffer against negative psychological outcomes.

Taken together, paternal overprotection shows a dyslexia-specific moderating effect, while emotional warmth and character strengths confer general benefits that apply equally to children with and without dyslexia. This distinction has important implications for intervention: programs targeting overprotective parenting may need to be tailored for families of children with dyslexia, whereas universal programs promoting emotional warmth, and character strengths engagement are likely to benefit all children.

## Limitations

Several limitations should be considered. First, given the cross-sectional design, the findings should be interpreted as associations rather than causal relationships. Second, the substantial case – control imbalance (only 5.9% of the sample had dyslexia) may have reduced statistical power to detect higher-order interactions, although our main interaction effect (Dyslexia × Paternal overprotection) was significant despite this imbalance. Third, the reliance on self-report data may introduce reporting bias and shared method variance; thus, incorporating teacher and observational assessments in future studies would be valuable. Fourth, our screening procedure identified dyslexia risk as a binary classification using questionnaires rather than individually administered reading achievement tests. Accordingly, findings relate to screening-defined dyslexia rather than clinical diagnosis, and future studies should incorporate continuous measures of reading severity. Fifth, the study sample was region-specific, limiting broader generalisability. Finally, while the current model focused on family and individual factors, school-level variables such as teacher support or classroom climate warrant further investigation to capture the full ecological context of children’s resilience.

## Conclusion

This study demonstrates that the impact of dyslexia on mental health is shaped not only by literacy difficulties but also by the interplay of family and individual factors. Using a moderation framework, we identified that paternal overprotection specifically moderates the dyslexia–depression relationship, while emotional warmth and character strengths confer general protective effects. These insights contribute to advancing educational psychology by linking literacy-related challenges to broader socioemotional development and provide practical directions for supporting children with learning difficulties in under-recognised contexts.

## Data Availability

Materials and analysis code for this study are available by emailing the corresponding author or the first author.

## References

[cit0001] American Psychiatric Association. (2013). *Diagnostic and statistical manual of mental disorders: DSM-5*. 10.1176/appi.books.9780890425596

[cit0002] Arrindell, W. A., Sanavio, E., Aguilar, G., Sica, C., Hatzichristou, C., Eisemann, M., Recinos, L. A., Gaszner, P., Peter, M., Battagliese, G., Kallai, J., & Ende, J. V. D. (1999). The development of a short form of the EMBU: Its appraisal with students in Greece, Guatemala, Hungary and Italy. *Personality & Individual Differences*, 27(4), 613–10. 10.1016/S0191-8869(98)00192-5

[cit0003] Asbari, M., Nurhayati, W., & Purwanto, A. (2020). The effect of parenting style and genetic personality on children character development. *Jurnal Penelitian dan Evaluasi Pendidikan*, 23(2), 206–218. 10.21831/pep.v23i2.28151

[cit0004] Bowlby, J. (1988). *A secure base: Parent-child attachment and healthy human development*. Basic Books.

[cit0005] Cai, L., Chen, Y., Hu, X., Guo, Y., Zhao, X., Sun, T., Wu, Y., & Li, X. (2020). An epidemiological study on the children with Chinese developmental dyslexia. *Journal of Developmental & Behavioral Pediatrics*, 41(3), 203–211. 10.1097/DBP.0000000000000751 31764410

[cit0006] Feng, W., Chotipanvithayakul, R., & Liu, H. (2024). Prevalence of dyslexia related to mental health problems and character strengths among primary school students in Northwest China. *Australian Journal of Psychology*, 76(1). 10.1080/00049530.2024.2399114 PMC12218475 40666644

[cit0007] Hou, F., Qi, L., Liu, L., Luo, X., Gu, H., Xie, X., Li, X., Zhang, J., & Song, R. (2018). Validity and reliability of the dyslexia checklist for Chinese children. *Frontiers in Psychology*, 9, 1–7. 10.3389/fpsyg.2018.01915 30356735 PMC6189409

[cit0008] Jing, J., Yu, M., & Deng, G. (1995). A comprehensive revision of the learning disability screening scale: Methodology and trial among primary school students [in Chinese]. *Psychological Development and Education*, 11(2), 24–29.

[cit0009] Kovacs, M. (2015). Children’s depression inventory (CDI and CDI 2). In R. L. Cautin & S. O. Lilienfeld (Eds.), *The Encyclopedia of Clinical Psychology* (pp. 1–5). John Wiley & Sons 10.1002/9781118625392.wbecp419

[cit0010] Lauritsen, J. M., Bruus, M., & Myatt, M. (2003). EpiData. *A Comprehensive Tool for Validated Entry and Documentation of Data*, 3, 98. http://www.epidata.dk

[cit0011] Li, D., Hu, K., Chen, G., Jin, Y., & Li, M. (1988). Shanghai urban area trial assessment report of the Raven’s progressive matrices combined-type (CRT) [in Chinese]. *Journal of Psychological Science*, 4, 27–31. 10.16719/j.cnki.1671-6981

[cit0012] Matthey, S., & Petrovski, P. (2002). The children’s depression inventory: Error in cutoff scores for screening purposes. *Psychological Assessment*, 14(2), 146–149. 10.1037//1040-3590.14.2.146 12056076

[cit0013] Mikulincer, M., & Shaver, P. R. (2007). *Attachment in adulthood: Structure, dynamics, and change*. Guilford Press.

[cit0014] Miller, L., Niemiec, R., McGrath, R., Tomasulo, D., Lipson, J., Bates, T. F. F., & Mayerson, N. (2021). *VIA Institute on Character–VIA Youth-103 (age 8–12)*. Retrieved December 12, 2021, from https://www.viacharacter.org/researchers/assessments/via-youth-103-8-12

[cit0015] Park, N., & Peterson, C. (2006). Moral competence and character strengths among adolescents: The development and validation of the values in action inventory of strengths for youth. *Journal of Adolescence*, 29(6), 891–909. 10.1016/j.adolescence.2006.04.011 16766025

[cit0016] Peterson, C., & Seligman, M. E. P. (2004). *Character strengths and virtues: A handbook and classification*. American Psychological Association; Oxford University Press.

[cit0017] Peterson, R. L., & Pennington, B. F. (2012). Developmental dyslexia. *Lancet*, 379(9830), 1997–2007. 10.1016/S0140-6736(12)60198-6 22513218 PMC3465717

[cit0018] Proyer, R. T., Gander, F., Wellenzohn, S., & Ruch, W. (2015). Strengths-based positive psychology interventions: A randomized placebo-controlled online trial on long-term effects for a signature strengths- vs. a lesser strengths-intervention. *Frontiers in Psychology*, 6(456), 14. 10.3389/fpsyg.2015.00456 25954221 PMC4406142

[cit0019] Qin, C., Cheng, X., Huang, Y., Xu, S., Liu, K., Tian, M., Liao, X., Zhou, X., Xiang, B., Lei, W., & Chen, J. (2022). Character strengths as protective factors against behavior problems in early adolescent. *Psicologia: Reflexão e Crítica*, 35(1), 11. 10.1186/s41155-022-00217-z PMC9156651 35641705

[cit0020] Shek, D. T., & Lee, T. Y. (2007). Parental behavioral control in academic and non-academic domains: A three-year longitudinal study in the Chinese culture. *International Journal of Adolescent Medicine and Health*, 19(4), 529–537. 10.1515/ijamh.2007.19.4.529 18348428

[cit0021] Su, L., Wang, K., Fan, F., Su, Y., & Gao, X. (2008). Reliability and validity of the Screen for Child Anxiety Related Emotional Disorders (SCARED) in Chinese children. *Journal of Anxiety Disorders*, 22(4), 612–621. 10.1016/j.janxdis.2007.05.011 17628391

[cit0022] Thompson, R. A. (1994). Emotion regulation: A theme in search of definition. *Monographs of the Society for Research in Child Development*, 59(2–3), 25–52. 10.2307/1166137 7984164

[cit0023] Waters, L. (2011). A review of school-based positive psychology interventions. *The Australian Educational and Developmental Psychologist*, 28(2), 75–90. 10.1375/aedp.28.2.75

[cit0024] Wen, W., Zhang, X., Wu, K., Guan, L., Huang, A., Liang, Z., Yu, X., Gu, Q., & Huang, Y. (2024). Association between parenting styles and dyslexia in primary school students: The mediating role of home literacy environment. *Frontiers in Psychology*, 15(1382519), 1–14. 10.3389/fpsyg.2024.1382519 PMC11209994 38939228

[cit0025] Xiao, P., Zhu, K., Liu, Q., Xie, X., Jiang, Q., Feng, Y., Wu, X., Tang, J., & Song, R. (2022). Association between developmental dyslexia and anxiety/depressive symptoms among children in China: The chain mediating of time spent on homework and stress. *Journal of Affective Disorders*, 297, 495–501. 10.1016/j.jad.2021.10.120 34743962

[cit0026] Yang, L., Li, C., Li, X., Zhai, M., An, Q., Zhang, Y., Zhao, J., & Weng, X. (2022). Prevalence of developmental dyslexia in primary school children: A systematic review and meta-analysis. *Brain Sciences*, 12(2), 26. 10.3390/brainsci12020240 PMC8870220 35204003

